# The Response of Estuarine Ammonia-Oxidizing Communities to Constant and Fluctuating Salinity Regimes

**DOI:** 10.3389/fmicb.2020.574815

**Published:** 2020-11-26

**Authors:** João Pereira Santos, António G. G. Sousa, Hugo Ribeiro, Catarina Magalhães

**Affiliations:** ^1^Centro Interdisciplinar de Investigação Marinha e Ambiental (CIIMAR), Universidade do Porto, Terminal de Cruzeiros do Porto de Leixões, Matosinhos, Portugal; ^2^Department F.A. Forel for Environmental and Aquatic Sciences, Section of Earth and Environmental Sciences, Institute for Environmental Sciences, University of Geneva, Geneva, Switzerland; ^3^Abel Salazar Institute of Biomedical Sciences, University of Porto (ICBAS-UP), Porto, Portugal; ^4^Faculdade de Ciências, Universidade do Porto, Porto, Portugal; ^5^School of Science & Engineering, University of Waikato, Hamilton, New Zealand; ^6^Ocean Frontier Institute, Dalhousie University, Halitax, NS, Canada

**Keywords:** salinity, nitrification (*amoA* AOA, *amoA* AOB), ^15^N isotope, 16S rRNA gene, AOB and AOA, estuarine communities

## Abstract

Aerobic nitrification is a fundamental nitrogen biogeochemical process that links the oxidation of ammonia to the removal of fixed nitrogen in eutrophicated water bodies. However, in estuarine environments there is an enormous variability of water physicochemical parameters that can affect the ammonia oxidation biological process. For instance, it is known that salinity can affect nitrification performance, yet there is still a lack of information on the ammonia-oxidizing communities behavior facing daily salinity fluctuations. In this work, laboratory experiments using upstream and downstream estuarine sediments were performed to address this missing gap by comparing the effect of daily salinity fluctuations with constant salinity on the activity and diversity of ammonia-oxidizing microorganisms (AOM). Activity and composition of AOM were assessed, respectively by using nitrogen stable isotope technique and 16S rRNA gene metabarcoding analysis. Nitrification activity was negatively affected by daily salinity fluctuations in upstream sediments while no effect was observed in downstream sediments. Constant salinity regime showed clearly higher rates of nitrification in upstream sediments while a similar nitrification performance between the two salinity regimes was registered in the downstream sediments. Results also indicated that daily salinity fluctuation regime had a negative effect on both ammonia-oxidizing bacteria (AOB) and ammonia-oxidizing archaea (AOA) community’s diversity. Phylogenetically, the estuarine downstream AOM were dominated by AOA (0.92–2.09%) followed by NOB (0.99–2%), and then AOB (0.2–0.32%); whereas NOB dominated estuarine upstream sediment samples (1.4–9.5%), followed by AOA (0.27–0.51%) and AOB (0.01–0.23%). Analysis of variance identified the spatial difference between samples (downstream and upstream) as the main drivers of AOA and AOB diversity. Our study indicates that benthic AOM inhabiting different estuarine sites presented distinct plasticity toward the salinity regimes tested. These findings help to improve our understanding in the dynamics of the nitrogen cycle of estuarine systems by showing the resilience and consequently the impact of different salinity regimes on the diversity and activity of ammonia oxidizer communities.

## Introduction

Nitrification, the oxidation of ammonium (NH_4_^+^) to nitrite (NO_2_^–^), and subsequently to nitrate (NO_3_^–^), plays an important role in the nitrogen (N) cycle (Kowalchuk and Stephen, 2001; Prosser and Embley, 2002). This process represents a critical link between mineralization of organic matter and the loss of fixed N via coupled nitrification–denitrification (Marchant et al., 2016; Zhu et al., 2018) and nitrification–anammox (Fernandes et al., 2016), being responsible for the removal of anthropogenic N in estuarine and marine environments. Nowadays, three functional groups of nitrifying prokaryotes have been identified based on their capacity to perform the nitrification process. The first group, which oxidizes ammonia or ammonium to nitrite (first step of the nitrification), are represented by the ammonia-oxidizing bacteria (AOB) with species within the β- and γ-*Proteobacteria* classes (Purkhold et al., 2000; Junier et al., 2010), and the ammonia-oxidizing archaea (AOA) represented by species affiliated with the *Thaumarchaeota* phylum (Stahl and de la Torre, 2012). The second group of microorganisms oxidizes nitrite to nitrate (second step of the nitrification), hence being identified as the nitrite-oxidizing bacteria (NOB). NOB are a phylogenetically heterogeneous group with species within the *Proteobacteria* (classes Alpha, Beta, and Gamma), *Nitrospirae*, *Nitrospinae*, and *Chloroflexi* (Daims et al., 2016). The third group is characterized by species able to perform the complete oxidation of ammonia to nitrate also known as comammox (Daims et al., 2015; van Kessel et al., 2015), with species within the *Nitrospirae* phylum (Lawson and Lucker, 2018).

Several studies have demonstrated that both archaeal and bacterial ammonia oxidizers are present in estuarine sediments (Magalhaes et al., 2007; Mosier and Francis, 2008; Monteiro et al., 2017; Zhu et al., 2018), with some areas dominated by AOA (Bernhard et al., 2010) and others by AOB (Li et al., 2015). Differences between AOA and AOB ratios have been linked to the elevated variability in terms of hydrodynamic, physical and chemical conditions observed in estuarine and intertidal sediments (Magalhães et al., 2009; Monteiro et al., 2017). In fact, key environmental factors thought to impact the distribution and activity of ammonia-oxidizing prokaryotes have been reported, and includes nitrogen forms (Dang et al., 2010; Sudarno et al., 2011), temperature (Sahan and Muyzer, 2008; Zeng et al., 2014), oxygen (Liu and Wang, 2015), pH (Guo et al., 2017), salinity (Bernhard et al., 2007; Zhang et al., 2015c; Santos et al., 2018), anthropogenic pollutants, like heavy metals (Beddow et al., 2016) among others. In the particular case of salinity, the high gradients of salinity along the estuarine areas have been shown to affect the magnitudes of nitrification rates (Berounsky and Nixon, 1993; Bernhard et al., 2007) by playing a major role in controlling NH_4_^+^ adsorption capacity of the sediment (Rysgaard et al., 1999; Weston et al., 2010). Also, salinity limits the distribution and functionality of the ammonia oxidizers by affecting their activity (Santos et al., 2018), abundance (Li et al., 2015), and diversity (Zhang et al., 2015c). Several studies have shown spatial differences in terms of nitrification activity in estuarine systems, with downstream communities displaying higher tolerance to salinity in comparison with upstream communities (Bernhard et al., 2007; Wang and Gu, 2014; Zhang et al., 2015c; Monteiro et al., 2017; Santos et al., 2018).

In terms of the key players involved in the nitrification process, previous studies showed some contradictory results in terms of AOA/AOB ratios across salinity gradient (Santoro et al., 2008; Bernhard et al., 2010; Li et al., 2015), suggesting the action of other parameters in ruling the prevalence of AOA or AOB (e.g., NH_4_^+^ levels). Despite the efforts to understand the dynamics of this process in estuarine systems, it is still unclear how the environmental parameters along the estuarine gradient control ammonia oxidizers diversity and activity. For instance, there is a lack of information about how the effect of short-term salinity fluctuation, experienced in estuarine systems, will influence the ammonia oxidation and the organisms that mediate this transformation. Yet, estuarine ammonia-oxidizing microorganisms (AOM) are exposed to different magnitudes of daily salinity fluctuations (Giblin et al., 2010; Yan et al., 2015), which varies according to the estuary location (e.g., upper estuary has low salinity variation compared with lower estuary).

In this study, we isolated through a controlled laboratory experiment, the effect of different salinity regimes (constant and daily fluctuation) on the activity and diversity of AOM from upstream and downstream estuarine sediments. We hypothesize that AOM sediments from upstream (Crestuma) and downstream (Afurada) estuarine locations would display distinct resilience toward short term salinity fluctuations.

## Materials and Methods

### Site Description and Sample Collection

The Douro River estuary is a mesotidal estuary with 21.6 km long located on the northwest coast of Portugal (Vieira and Bordalo, 2000). It is located in a highly urbanized area, promoting several anthropogenic activities, and consequently the increase of pollutants in water and sediments (Madureira et al., 2010; Cruzeiro et al., 2017; Ribeiro et al., 2018). In July 2015, intertidal sediments from two sites (Afurada and Crestuma) along the salinity gradient of the Douro River estuary were collected. Afurada (AF) is located in the downstream estuary, being characterized by a highly dynamic site with marked daily salinity changes due to the influence of coastal Atlantic saltwater. Crestuma (CR) is located in the upstream estuary and highly influenced by freshwater riverine input. Information about the *in situ* physical and chemical characteristics of Douro River estuary and more precisely Afurada and Crestuma locations have been reported previously in other studies (Monteiro et al., 2017; Santos et al., 2018).

Surface sediments (2 cm depth) were randomly collected from 10 sub-locations of each site (approximately 5 kg), homogenized in sterile plastic boxes (10 L) and stored in refrigerated ice chests. In the laboratory, sub-samples of homogenized sediment were collected into sterile plastic bags (VWR Sterile Sample Bags) and stored at −80°C for later DNA extraction, while other sub-samples were used to set up the different salinity regimes experiments. Water used for the incubation experiment was collected from the Douro River (around 1200 L) using a firetruck and stored in appropriated and cleaned tanks at CIIMAR.

### Experimental Setup

Experiments were designed to simulate different salinity regimes which consisted of two salinity treatments *per* site (AF and CR) in triplicate. The two salinity regimes consisted of a constant salinity treatment (with salinity at 0 psu for CR (upstream) sediments and with salinity at 15 psu for AF (downstream) sediments) based on the predominant site salinity characteristics (Santos et al., 2018), and a salinity fluctuation treatment which consisted on gradual daily oscillations between salinities of 0, 15, and 30 psu. The sediments subjected to a constant salinity regime of 0 and 15 psu were labeled as CR_C and AF_C, respectively for CR and AF sediment reactors. The AF and CR sediments under the salinity fluctuation regime were labeled as CR_Δ and AF_Δ, respectively.

An autonomous water agitation system was built with 12 acrylic reactors, each with 14 cm diameter and 33 cm height (Supplementary Figure S1), with a rotation set at 32 rpm to maintain water homogenized and oxygenized. The photoperiod was simulated, starting at 7:30 am and switching off at 7:30 pm. All materials in contact with water and sediments were acid cleaned (10% HCl) before the beginning of the experiment. The sediments from upstream (CR) and downstream (AF) locations were added to six reactors (final volume of 708.47 cm^3^, 10 cm depth) in a total of 12 reactors. Douro River water (1.2 L) adjusted to the desired salinities (0, 15, and 30 psu) with marine salts (Tropic Marin) and supplemented with 20 μM of NH_4_Cl was also added to each reactor. Every 12 h, this water (1.2 L) was renewed in each treatment (constant and fluctuation) with its respective salinity and NH_4_Cl supplementation. The water used for the incubations had a pH of 8.14, and the following nutrients concentrations: 111.3 μM of NO_3_^–^, 0.1 μM of NO_2_^–^ and 0.1 μM of NH_4_^+^/NH_3_. Salinity and water temperature were monitored before water renewal using a conductivity meter CO 310 (VWR Collection).

In the 30^th^ day of incubation, the renewed 1.2 L water was supplemented with 20 μM of labeled [^15^N]H_4_Cl to measure nitrification rates directly in the reactors (Denk et al., 2017; Xia et al., 2017; Jäntti et al., 2018). Site sediment treatments had the same salinity (Afurada treatments = 15 psu and Crestuma treatments = 0 psu) when exposed to labeled ^15^NH_4_Cl.

After 36 days of incubation, composed surface sediment samples (2 cm depth) from the triplicate reactors (CR_C, CR_Δ, AF_C, and AF_Δ) were also collected to measure nitrification activity by performing slurries incubations. For those measurements both CR sediments were incubated with water at salinity of 0 psu, while AF sediments were incubated with salinity at 15 psu. To each 100 mL serum bottle, it was added 35.3 ± 1.2 g of wet weight of sediment. Water (50 mL) was amended with 100 μM of [^15^N]H_4_^+^ to measure nitrification rates in the different conditions (constant vs. fluctuation).

In both cases, 8 mL of overlying water was collected from each slurry after 2 h of incubation, centrifuged at 420 × *g* (Compact Star CS4), filtered at 0.2 μm (VWR Syringe Filters) and stored at −20°C for later isotopic analyses. During incubation, serum flasks were held in a dark room at constant temperature (20°C) with constant agitation at 100 rpm.

### Chemical Analysis

#### Nitrification Rates

Potential nitrification rates were measured using the stable isotope technique, where samples were amended with [^15^N]H_4_^+^ and the production of [^15^N]O_2_^–^ and [^15^N]O_3_^–^ were measured, giving an estimate of the potential uncoupled nitrification occurring in the water. The analyses were determined using the denitrifier method (Sigman et al., 2001) at Appalachian Lab, University of Maryland. Potential nitrification rates were calculated based on the [^15^N] fractional change (R$\frac{15}{14}$N) after 2 h of incubation, and the [NO_3_^–^] and [NO_2_^–^] existent at the same period, using the equation described by Jantti et al. (2012).

N[μmolNxh-x]-1=(([NO+3-NO]2-×vol)/

$$\mathrm{weigth}\mathrm{or}\mathrm{area})\times \text{R}\frac{15}{14}\text{N})/\Delta t$$

where N correspond to the potential uncoupled nitrification, [NO_3_^–^ + NO_2_^–^] is the NO_3_^–^ and NO_2_^–^ concentration at the end of the incubation (μM), vol is the volume of water in the system (L), weight is the weight of sediment in the slurries (*x* = g) and area is the area corresponding to the reactors (*x* = cm^2^), $\text{R}\,\frac{15}{14}\,\text{N}$ is the ratio between atom 15 and atom 14 in the samples after 2 h of incubation, Δt is the incubation time (2 h).

### Molecular Analysis

#### Nucleic Acid Extraction and Quantification

Total DNA was extracted from 0.5 g wet weight of homogenized sediment (from *in situ* AF and CR sediments, as well as from each reactor at the end of the salinity regimes experiment) using the PowerSoil DNA isolation kit (MoBio Laboratories Inc., Solana Beach, CA, United States). Similarly, total RNA was extracted from 2 g wet weight of homogenized sediment from each reactor at the end of the salinity regimes experiment using the Power Soil RNA isolation kit (MoBio Laboratories Inc.) according to MoBio instructions. DNA was digested from the RNA pool by being treated with 1 U μL^–1^ of DNase I (Sigma) using DNA- and RNA-free reagents, followed by PCR using general 16S rRNA gene bacterial primers to check for traces of genomic DNA contamination. Reverse transcription (RT) was performed using the Omniscript RT kit (Qiagen) by adding 13 μL of total RNA to an 18 μL RT mixture following the manufacturer’s instructions. The DNA and cDNA were quantified by the Qubit^®^ dsDNA HS assay and Qubit^®^ ssDNA Kit (Life Technologies), respectively, according to manufacturer’s instructions.

#### Next-Generation Sequencing of the 16S rRNA Gene Amplicons

DNA extracted from sediment samples from AF and CR at the beginning of the experiment (AF_i and CR_i) as well as from two of the triplicate reactors subjected to the different salinity regimes (AF_C, CR_C, AF_△, and CR_△) was used for sequencing the 16S rRNA gene. The primer set 515F-Y (5′-GTGYCAGCMGCCGCGGTAA-3′) (Caporaso et al., 2012) and 926R (5′-CCGYCAATTYMTTTRAGTTT-3′) (Parada et al., 2016) targeting the V4-V5 region of the archaeal and bacterial 16S rRNA genes was used to assess the whole prokaryotic community diversity (Wear et al., 2018). This primer set yielded accurate estimates of complex prokaryotic mock communities increasing the *Thaumarchaeota* coverage relatively to other primer sets (Parada et al., 2016). Although 16S rRNA primers will not cover the entire diversity of ammonia oxidizers, the monophyly of most of AOA and AOB groups facilitate the use of 16S rRNA gene to study the diversity of ammonia oxidizers, being its phylogeny mostly congruent with *amoA* gene phylogeny (Alves et al., 2018). Amplicons were used to build Illumina paired-end libraries (2 × 300 bp) sequenced on an Illumina MiSeq platform using V3 Chemistry (Illumina). These steps were carried out by LGC Genomics (LGC Genomics GmbH, Berlin, Germany).

#### PCR Amplification and Denaturing Gradient Gel Electrophoresis (DGGE)

PCR amplification of β-proteobacterial *amoA* genes and transcript fragments were performed using the primer set amoA-1F (5′-GGGGTTTCTACTGGTGGT) with a GC clamp (CGCCCGCCGCGC CCCGCGCCCGGCCCGCCGCCC CCGCCCC) (Muyzer et al., 1993) and amoA-2R (5′-CC CCTCKGSAAAGCCTTCTTC) (Rotthauwe et al., 1997). For the amplification of fragments of archaeal *amoA*, it was used the primer set CrenamoA23f (5′-ATGGTCTGGCTWAGACG) and CrenamoA616r (5′-GCCATCCATCTGTATGTCCA) (Tourna et al., 2008). All conditions used were the same described in Santos et al. (2018).

The denaturing gradient gel electrophoresis (DGGE) technique was performed using a DCode Universal Mutation Detection System (Bio-Rad, Hertfordshire, United Kingdom). The conditions were established according to Yamamoto et al. (2010). Afterward, gels were silver stained based on Karpouzas and Singh (2010) and scanned using a GS-800 Calibrated Densitometer (Bio-Rad, Hertfordshire, United Kingdom). The DGGE gel images were converted to a densitometry scan and aligned using image analysis software (Quantity One software). The software records a density profile through each DGGE lane, detects the bands, and calculates the relative contribution of each band to the total band signal in the lane after applying a rolling disk function as background subtraction. Then, values corresponding to the band position were imported into the Primer 6 software package (version 6.1.11) (Clarke and Gorley, 2006). Based on the presence/absence of individual bands in each lane, a binary matrix was created using Primer 6 and hierarchical cluster analysis based on Bray–Curtis similarity (Clarke and Warwick, 2001). DGGE results and the respective gel profiles images can be found in Supplementary Figures S2–S6.

### Data Analysis

#### Statistical Analysis

Data from the nitrification rates were analyzed in triplicate for all relevant parameters. Data were tested for normality using the Kolmogorov–Smirnov test, and for homoscedasticity using Levene’s test. In both tests the null hypothesis was not rejected (*P*-value > 0.5) meaning that the data is not statistically different from a normal distribution with equal variance across the groups being compared. Significant differences between pairwise means were assessed using *t*-tests. All analyses were performed at the 95% confidence level and the statistical tests were performed using the software STATISTICA (version 13, StatSoft, Inc.).

#### Bioinformatic Analysis of NGS 16S rRNA Gene Amplicons

16S rRNA gene amplicons from different samples were sequenced in three different sequencing runs: run 1 (samples AF_C1, AF_C2, AF_Δ2, CR_C2, CR_Δ2), run 2 (AF_Δ1, CR_C3, CR_Δ1), and run 3 (AF_i and CR_i). Primer clipped forward and reverse fastq files were imported, filtered, truncated, trimmed, merged and denoised using DADA2 package (v.1.14.0) (Callahan et al., 2016) implemented in R (v.3.4.1) (R Core Team, 2017). Forward and reverse reads were truncated according to the quality of each sequencing run: 200 and 190 bp for the run 1, 200 and 200 bp for the run 2, and 220 and 220 bp for the run 3. Reads with ambiguities (i.e., N) were discarded and filtered with a truncation quality score of two and maximum expected error of eight for both reads. Then, forward and reverse fastq reads were dereplicated into unique reads and pooled independently for each run to estimate the error rates using the default 1e^8^ number of bases (randomized) and to denoize sequences into Amplicon Sequence Variants (ASV). The denoised unique forward and reverse reads were merged using the default parameters (without allowing mismatches and with a minimum overlap of 12 bp) and the sequences out of the target range (367–377 bp) were discarded. Chimeras were removed using the consensus method implemented in the DADA2 R package (v.1.14.0). Finally, the ASVs were taxonomically classified against the SILVA SSU Ref NR database (v.132) (Quast et al., 2013) using the RDP Naive Bayesian Classifier algorithm (Wang et al., 2007; Callahan, 2018) with a minimum bootstrap confidence for assigning a taxonomic level of 60%. Additionally, the ASVs that matched exactly with one reference fasta sequence within the SILVA database were classified until the species level.

The ASVs fasta sequences were imported to QIIME2 (v.2018.4) (Bolyen et al., 2019) to use the available plugins to build a phylogenetic tree. First, MAFFT (v.7) (Katoh and Standley, 2013) plugin was used to perform a multiple sequence alignment (MSA). The MSA was masked to eliminate the highly variable positions and the masked MSA was used to build a maximum-likelihood tree with FastTree 2 (Price et al., 2010) plugin. Ultimately, the phylogenetic tree was rooted through the midpoint rooting method.

The ASV table, taxonomy table and phylogenetic tree were imported to the phyloseq R package (v.1.28.0) (McMurdie and Holmes, 2013) in order to perform beta-diversity analyses. Whereas the beta-diversity metrics estimated were the unweighted and weighted UniFrac (Lozupone and Knight, 2005; Lozupone et al., 2007) visualized through the Principal Coordinate Analysis (PCoA) method. For beta-diversity analysis, the compositional table of ASVs was transformed in the following way: ASVs that appeared less than six times, in less than three samples were excluded; sequence reads *per* sample were transformed to counts *per* million; and kept only the top 10 phyla (*Proteobacteria, Bacteroidetes, Planctomycetes, Cyanobacteria, Acidobacteria, Nitrospirae, Thaumarchaeota, Gemmatimonadetes, Verrucomicrobia, and Chloroflexi*). This represents in average *ca.* 85% of reads in relation to the initial number of sequences. The final plots were visualized and compiled using the ggplot2 (v.3.3.0) (Wickham, 2009) and the gridExtra (v.2.3) (Auguie and Antonov, 2017) R packages, respectively. Multivariate homogeneity of groups dispersions and permutational multivariate analysis of variance of unweighted and weighted UniFrac distance matrices were performed to assess the within- and between-group variation with the *betadisper*() and *adonis()* functions from the vegan (v. 2.5.6) R package (Oksanen et al., 2018). It was only rejected the null hypothesis of the second test, when the null of the first was not rejected, i.e., only when there was not found statistically significant differences within-group variation, it was tested between-group variation hypothesis.

Nitrifying ASVs were retrieved by searching the “*Nitro*” prefix throughout all the taxonomic levels (from phylum to genus). Then, the putative nitrifying taxa were manually checked. The ASV_3095 and ASV_6335 classified as *Methylophaga* (genus), the ASV_3517 as *Leptospirillum* (genus) and ASV_6835 as *Thermodesulfovibrionia* (until class) were removed because do not constitute potential nitrifying taxa and due to the lack of confidence. In addition, all unclassified ASVs at genus level or environmental clones/sequences were also removed due to the lack of confidence.

Sequences data sets from 16S rRNA amplicon used in this study were deposited in the European Nucleotide Archive under the project accession number PRJEB38114.

## Results

### Experimental System

Sediments exposure to different salinity regimes was performed at room temperature (18.8 ± 2.2°C) during 36 days. Water temperature and salinity measurements from the different reactors (AF_C, CR_C, AF_Δ, and CR_Δ) are presented in Supplementary Table S1. Salinity was maintained steady, with minor variations in constant treatments triplicate reactors (AF_C and CR_C). On salinity fluctuation treatments (AF_Δ and CR_Δ), the salinity ranged between 1.2 and 29.3 psu in the upstream sediments reactors (Crestuma) and between 1.5 and 29.3 psu in the sediment reactors from downstream station (Afurada).

### Potential Nitrification Rates

**FIGURE 1:**
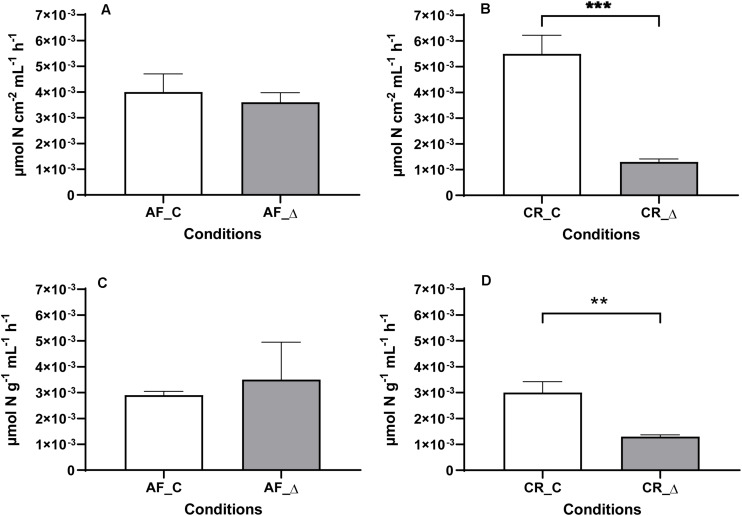
Mean and standard deviation (*n* = 3) of potential nitrification rates ([^15^N]O_2_^–^ + [^15^N]O_3_^–^ produced) measured in the sediments subjected to daily salinity fluctuations (AF_Δ and CR_Δ) and constant salinity (AF_C and CR_C) treatments. Rates were measured with the same salinity (0 psu for Crestuma sediments, and 15 for Afurada sediments). Nitrification rates are displayed in four different charts corresponding to Afurada (AF) reactors **(A)** and Crestuma (CR) reactors **(B)** after addition of 20 μM of [^15^N]H_4_^+^), and Afurada slurries **(C)** and Crestuma slurries **(D)** after addition of 100 μM of [^15^N]H_4_^+^). Symbol ** and *** means a *P* < 0.01 and *P* < 0.001, respectively.

Nitrification rates in constant (AF_C and CR_C) and fluctuation (AF_Δ, CR_Δ) conditions were estimated directly in the reactors (Figures 1A,B) as well as in sediment slurries incubations (Figures 1C,D). Nitrification measurements in the reactors and in sediment slurries were comparable, with estuarine downstream sediments (AF) presenting similar (*t*-test, *P* ≥ 0.05) nitrification rates between the two salinity regimes tested (AF_C and AF_Δ) (Figures 1A,C). In contrast, nitrification rates under the constant salinity regime in the upstream site (CR) were significantly (*t*-test, *P* < 0.05) higher than those subjected to the fluctuating salinity regime (CR_Δ). Again, these results were similar for direct reactors measurements and for slurries incubations (Figures 1B,D). Nitrification rates measured directly in the reactors and in slurries for each salinity treatment are presented in Supplementary Tables S2, S3, respectively.

### Denaturing Gradient Gel Electrophoresis of Archaeal and Bacterial *amoA* Genes and Transcripts

Fingerprinting analysis of the archaeal and bacterial *amoA* gene fragments (DNA) and transcripts (cDNA) of Afurada (AF_i, AF_Δ and AF_C) and Crestuma (CR_i, CR_Δ and CR_C) sediments are presented in Supplementary Figures S2, S3.

For the constant salinity treatment, results from the downstream sediments (AF) showed that DGGE bands of AOA genes and transcripts decreased compared to the initial samples (Figure S2A). However, in the salinity fluctuation treatment, the number of DGGE bands from AOA genes and transcripts remained similar to the initial samples (Figure S2A). In the upstream (CR) sediments higher number of DGGE bands of AOA genes were found in the constant salinity treatment compared with the salinity fluctuation regime (*t*-test, *P* < 0.05; Figure S2B). DGGE profiles of archaeal *amoA* gene transcripts were also more diverse in the constant salinity treatment compared with the salinity fluctuation regime.

In AF sediments, the DGGE profile (Figure S3A) of bacterial *amoA* gene showed differences (*t*-test, *P* < 0.05) between the number of DGGE bands of *amoA* gene fragments exposed to the different salinity regimes (AF_C and AF_Δ). However, similar (*t*-test, *P* ≥ 0.05) number of DGGE bands of AOB *amoA* transcripts was observed for AF_Δ treatment and constant salinity regime. In CR sediments, a clear increase (*t*-test, *P* < 0.05) of the DGGE bands of the bacterial *amoA* gene were observed between CR_i, CR_C, and CR_Δ. However, in CR sediments, the bacterial *amoA* gene transcripts were not detected (Figure S3B).

### Prokaryotic Community

The Illumina Miseq platform originated ∼680000 raw reads of the 16S rRNA gene (V4–V5) amplicons from the ten samples analyzed. After the quality filtering steps and chimera removal, it was obtained a total of 489414 16S rRNA gene amplicon sequences ranging from 19636 (AF_i sample) to 113330 (AF_Δ1 sample) sequences *per* sample. Due to the nature of Illumina sequencing, the number of obtained sequences varied among samples. A total of 98.3% good-quality sequences were taxonomically classified as Bacteria, 1.7% as Archaea and 0.0045% as Eukaryote.

**FIGURE 2:**
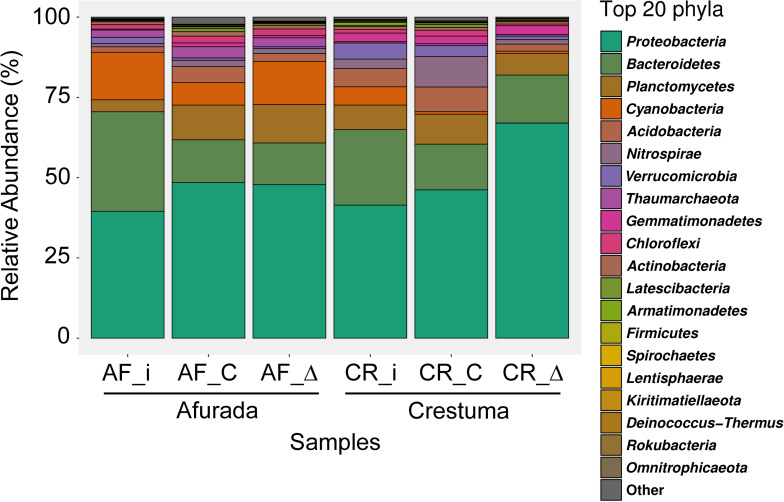
Bar plot representing the relative abundance of the top 20 most abundant prokaryotic phyla from the initial sediment (AF_i and CR_i) and from the sediment samples of the different salinity treatments (constant – AF_C and CR_C, and fluctuation – AF_Δ and CR_Δ). Samples under different salinity treatments were depicted as mean relative abundances of phyla from original sample replicates (*n* = 2).

The top 20 phyla are presented in Figure 2. *Proteobacteria* was the most abundant phylum in all samples analyzed. *Bacteroidetes*, *Cyanobacteria*, and *Verrucomicrobia* phyla relative abundance decreased in all treatments in comparison with initial sediment (AF_i and CR_i). *Planctomycetes* phylum relative abundance increased in AF treatments while CR treatments maintained a similar relative abundance with initial sediment. *Acidobacteria* phylum only increased in AF_C treatment while decreased in CR_Δ treatment in comparison with each respective initial sediment. The relative abundance of *Thaumarchaeota* phylum was similar across all treatments, with a considerable higher abundance in AF samples. Lastly, *Nitrospirae* phylum presents a tremendous increase in CR_C treatment in comparison with initial sediment and CR_Δ. Detailed information about the relative abundance of each phyla across all samples can be found in Supplementary Tables S4.

**FIGURE 3:**
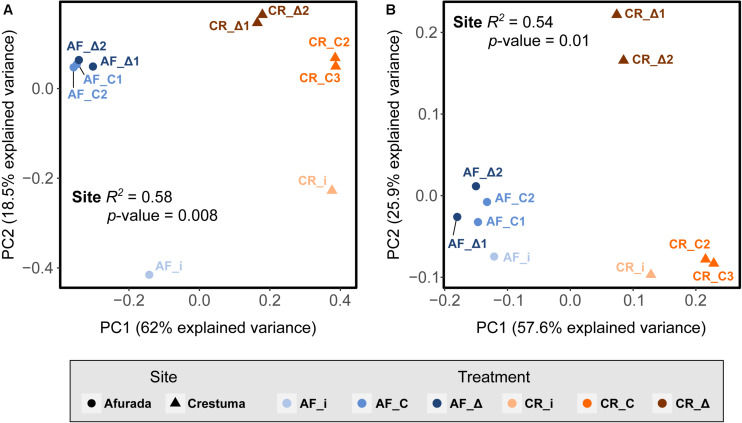
Principal Coordinates Analysis (PCoA) of **(A)** unweighted and **(B)** weighted UniFrac distances of ASVs belonging to the top 10 phyla from the initial sediment (AF_i and CR_i) as well as in the sediment samples of the different salinity treatments (constant – AF_C and CR_C, and fluctuation – AF_Δ and CR_Δ). The *R*^2^ and *P*-values that resulted from the Adonis analysis of unweighted and weighted UniFrac distance matrices grouped by site and treatment are highlighted inside the PCoA. Samples were grouped by site (Afurada – AF_i, AF_C1, AF_C2, AF_Δ1, AF_Δ2; and Crestuma – CR_i, CR_C2, CR_C3, CR_Δ1, CR_Δ2) and treatment (Afurada *in situ* sample – AF_i; Afurada constant salinity – AF_C1, AF_C2; Afurada fluctuation salinity – AF_Δ1, AF_Δ2; and Crestuma *in situ* sample – CR_i; Crestuma constant salinity – CR_C2, CR_C3; Crestuma fluctuation salinity – CR_Δ1, CR_Δ2).

The differences in the prokaryotic composition, across sediment samples from the salinity regimes tested (AF_i, CR_i, CR_C, AF_C, AF_Δ, and CR_Δ) were explored in the PCoA based on unweighted and weighted UniFrac distances (Figures 3A,B). The prokaryotic composition between replicates were found to be highly homogeneous (Figure 3) highlighting that despite some of the replicates have been sequenced in different runs, the data is highly reliable and comparable (e.g., CR_Δ2, run 1 and CR_Δ1, run 2). Principal coordinate 1 (PC1) explained the majority of the variance (62%) in the prokaryotic communities based on the presence/absence of the phylogenetic distance of the ASVs – unweighted UniFrac distance – belonging to the 10 most abundant phyla (Figure 3A). The variance explained by the abundance of the phylogenetic distance of the ASVs (of the top 10 phyla) – weighted UniFrac distance – was similar (Figure 3B). In both analyses, the permutational multivariate analysis of variance (permanova) found that site location (downstream and upstream stations) was a strong driver on differences in microbial composition (*R*^2^ = 0.58 and *P*-value = 0.008 and *R*^2^ = 0.54 and *P*-value = 0.01, Figures 3A,B).

### Taxonomic Composition of Nitrifying Prokaryotes

**FIGURE 4:**
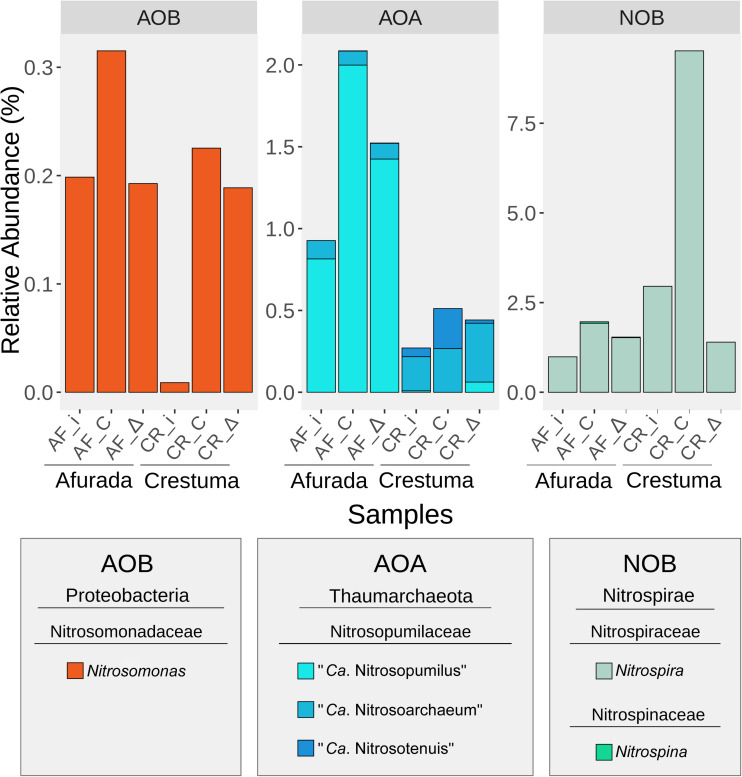
Bar plot representing the relative abundance of the identified nitrifying taxa at genus level in the initial sediment (AF_i and CR_i) as well as in the sediment samples of the different salinity treatments (constant – AF_C and CR_C, and fluctuation – AF_Δ and CR_Δ). Samples under different salinity treatments were depicted as mean relative abundances of genera from original sample replicates (*n* = 2).

The relative abundance of the nitrifying organisms at the genus level in all samples is presented in Figure 4. In the version 132 of SILVA SSU Ref NR database β-*Proteobacteria* class was rearranged under the *Betaproteobacteriales* order within the class γ-*Proteobacteria*. Therefore, what is known as AOB β- and γ*-Proteobacteria* in some taxonomies and literature, in 132 version of SILVA database is rearranged under the γ-*Proteobacteria* class, particularly within the family *Nitrosomonadaceae* (*Betaproteobacteriales* order). *Nitrosomonas* was the only AOB genus identified with higher relative abundance in the constant salinity treatments of AF and CR (Supplementary Figure S7 and Supplementary Table S5). Regarding the AOA taxonomic groups, one family was identified, the *Nitrosopumilaceae* which included representatives of “*Ca.* Nitrosopumilus”, “*Ca.* Nitrosoarchaeum”, and “*Ca.* Nitrosotenuis”.

“*Ca.* Nitrosopumilus” was found to be highly representative in AF sediments, while “*Ca.* Nitrosoarchaeum” and “*Ca.* Nitrosotenuis” are over-represented in CR sediments with a pronounced increase of “*Ca.* Nitrosotenuis” in the freshwater treatment (CR_C). Two genera belonging to the NOB group were also identified in the data set. While *Nitrospira* presented higher relative abundance in downstream sediments, *Nitrospina* showed an opposite pattern with the highest values registered at the constant salinity treatment in upstream sediments (Supplementary Figure S7 and Supplementary Table S5).

## Discussion

The objective of this study was to test whether upstream and downstream AOM communities would display different responses to salinity fluctuations. For that, we simulated different salinity regimes in controlled reactors with estuarine sediments and overlying water from the Douro River to mimic natural physiochemical conditions.

It can be assumed that laboratory experiments do not reflect the natural conditions, which are extremely dynamic and complex; however it can provide valuable insights of how short-term constant and salinity fluctuations can shape and affect AOM activity and diversity.

The downstream estuarine site (Afurada) is characterized by its marked daily salinity amplitudes [e.g., 3.4–14.0 psu variation in December 2014 (Santos et al., 2018)]. These amplitudes are expected to select communities more resilient to the natural salinity regime (Crump et al., 2004; Wang et al., 2018). Our results showed no significant differences between nitrification rates in downstream estuarine sediments exposed to constant salinity vs. fluctuating regime. Thus, suggesting that AOM from estuarine downstream sediments display an elevated plasticity to different salinity regimes which can be advantageous when inhabiting environments with high salinity amplitudes. Although, to the best of our knowledge, the effect of daily salinity fluctuations has not been tested directly, some previous works point that AOM communities from downstream estuarine systems displayed similar potential nitrification rates under salinities ranges of 15 and 30 psu (Magalhaes et al., 2005; Bernhard et al., 2007; Santos et al., 2018). Yet, it should be noted that within the AOM, AOB and AOA activity may differ in response to different salinities, as previously reported in other high salinity amplitude environments such as Weeks Bay (Caffrey et al., 2007), Douro River estuary (Magalhães et al., 2009), Plum Island Sound estuary (Bernhard et al., 2007, 2010), Huntington Beach subterranean estuary (Santoro et al., 2008), Cochin estuary (Puthiya Veettil et al., 2015), and Colne estuary (Li et al., 2015).

Prokaryotic beta diversity analysis showed a high similarity between Afurada *in situ* communities and the ones exposed to 36 days of constant and daily salinity fluctuations, highlighting the elevated resilience of the prokaryotic communities from Afurada site to salinity shifts. With primers able to capture archaeal and bacterial 16S rRNA genes simultaneously and with particularly good coverage and resolution for *Thaumarchaeota* (Parada et al., 2016), we were able to identify known genera of AOA and AOB. However, it is important to recognize that this analysis does not target specifically the 16S rRNA gene nor the functional genes of AOM communities. The prokaryotic composition analysis in Afurada treatments identified higher relative abundance of the AOA “*Ca.* Nitrosopumilus” genus in both constant and fluctuation salinity treatments in comparison with *in situ* sediments (2.0 and 1.4%, for AF_C and AF_Δ, respectively). In addition, relative low abundance of the other AOA genera (“*Ca.* Nitrosoarchaeum” and “*Ca.* Nitrosotenuis”) was registered in AF_C and AF_Δ treatments. The higher detection of “*Ca.* Nitrosopumilus” genus in both salinity treatments in comparison with the other two genera can be explained by the fact that *Nitrosopumilus* taxa has been often associated to saline environments (Bouskill et al., 2012; Xie et al., 2014). For instance, Jin et al. (2010) reported the dominance of *Nitrosopumilus maritimus*-like in a laboratory-scale bioreactor treating saline wastewater under 10 psu. Whereas, Wu et al. (2013) hypothesized that a high salinity condition could promote the growth of the marine group representative, *N. maritimus*. Similarly, Qin et al. (2017) reported *Nitrosopumilus* species capable of growing over a wide salinity range, suggesting even a possible adaptation to seasonal salinity fluctuations in estuarine environments, which is in agreement with our findings.

Concerning the AOB groups, only the *Nitrosomonas* genus was identified in our analysis with an increase (compared to initial *in situ* sediment – CR_i) in constant salinity and no difference in the salinity fluctuation, suggesting a good adaptation of Afurada AOB to the salinity regimes tested. After incubating sediments from Jiulong River during 28 days, Wang et al. (2018) reported that the abundance of AOB increased at intermediate salinity levels (ranging from 0, 10, 20, and 30 psu). The capacity of AOB to be more resilient in higher salinities compared to lower salinities has also been previously reported (Gonzalez-Silva et al., 2016; Zhou et al., 2017). Indeed, our targeted analysis of *amoA* gene fragments supported this assumption with high similarity between treatments and initial *in situ* sediments in the hierarchical cluster analysis.

The upstream estuarine site (Crestuma) is characterized by low salinity amplitudes (Azevedo et al., 2010), and thus, expected to promote the development of communities with low tolerance to high salinity amplitudes. As a consequence, the different experimental incubation conditions (constant salinity of 0 psu, similar to *in situ* conditions; and salinity fluctuations quite different from *in situ* conditions) would be expected to have a distinct impact in these community’s activity and structure. Indeed, significantly higher nitrification rates were detected in Crestuma constant salinity regime when compared with fluctuation regime. This is in agreement with the differences in the prokaryotic communities observed between the CR_Δ and CR_C treatments (see Figure 3). These results support our initial hypothesis that prokaryotic communities in the different sites are well established toward their *in situ* natural salinity regimes. Previously, Santos et al. (2018) exposed *in situ* Crestuma sediments to three constant salinities (0, 15, and 30 psu) and found a significant decrease in nitrification rates when sediments were exposed to higher salinities. Similarly, (Bernhard et al., 2007) found a significant nitrification rate decrease when exposing sediments (form upstream region) to high salinity (30 psu). In agreement Zhang et al. (2015c) through the incubation of estuarine sediments found that AOB showed the highest transcriptional activity in the low-salinity microcosms.

The AOA groups identified by the 16S rRNA gene sequencing analysis showed the appearance of the saline-associated “*Ca.* Nitrosopumilus” (Bouskill et al., 2012) on the fluctuation treatment suggesting a shift from the initial natural community. “*Ca.* Nitrosotenuis” was more abundant at the freshwater treatment (CR_C) as expected since previous studies identified this group as non-halophilic (Lebedeva et al., 2013; Li et al., 2016; Sauder et al., 2018). In the case of “*Ca.* Nitrosoarchaeum,” despite being associated with low salinity habitats (Mosier et al., 2012; Lebedeva et al., 2013), it was favored in both salinity conditions suggesting a certain tolerance of this genus to salinity fluctuations.

Differences on AOA phylotypes within the different salinity treatments, based on the 16S rRNA gene sequencing analysis, were also reflected in the fingerprinting *amoA* gene transcripts analysis that showed 30% of relative dissimilarity between the DGGE profile of AOA *amoA* gene transcripts of the different salinity treatments.

The AOB group identified in Crestuma sediments was also the *Nitrosomonas* genus, which increased after 36 days of incubation in both salinity treatments, although with higher relative abundance in the constant salinity treatment. The fingerprinting β-AOB *amoA* transcript analysis suggested an increase number of β-AOB phylotypes in both salinity treatments (mainly in the salinity fluctuation regime) since a higher detection of DGGE bands were registered in the experimental salinity treatments in comparison with the Crestuma *in situ* sediment. Yet, hierarchical cluster analysis of the β-AOB *amoA* DGGE bands profiles suggests that the β-AOB phylotypes of both salinity treatments are quite distinct and therefore could contribute to a certain extent to the different nitrification rates obtained. In fact, Cortés-Lorenzo et al. (2015) showed that the increase of concentrations of NaCl of 24.1 g L^–1^ in a freshwater water effluent resulted in a remarkable decrease of the NH_4_^+^ oxidation capacity of the system and a shift in AOB species present.

Changes in Crestuma community structure facing the different salinity regimes is likely to occur especially within the salinity fluctuation treatment due to the low *in-situ* salinity amplitudes that characterize Crestuma site (Santos et al., 2018), and thus the adaptation of its communities to lower osmotic pressures (Moussa et al., 2006; Jeffries et al., 2012).

Yet, the differences found between initial *in situ* communities and salinity treatments might suggest that other constant experimental factors could have played an important role in the results obtained. Indeed, studies have been shown the importance of salinity in estuarine salinity gradient environments (Zhang et al., 2015a; Gao et al., 2018; Li et al., 2018), however other factors have also been shown to contribute substantially to the shaping of the AOM communities – e.g., NH_4_^+^ levels (Verhamme et al., 2011; Zhang et al., 2015b; Gao et al., 2016). Thus, the addition of 20 μM of NH_4_Cl every 12 h could have played an important role in the development of AOM, such as AOB communities (under both salinity conditions), since Crestuma site is characterized by low ammonium concentrations in the water column.

The prokaryotic composition among the different incubated sediments suggests that comammox process, carried out by some *Nitrospira* species (Daims et al., 2015; Zhao et al., 2019) could have played an important role in the nitrification rates differences seen between Crestuma salinity regimes (CR_C and CR_Δ). This assumption is to a certain extent supported by the non-detection of AOB *amoA* transcripts (see Supplementary Figures S3, S6B) under Crestuma constant and salinity fluctuation treatments (CR_C and CR_Δ). Similar results were reported by Santos et al. (2018) and Monteiro et al. (2017) for sediments from the same Douro estuarine location collected within different years. Monteiro et al. (2017) not only found greater abundances of NOB at Crestuma site, but also a positive correlation with *Nitrospira* genus and the potential nitrification rates. In our study, we observed a substantial increase of *Nitrospira* genus from 2.95% (*in-situ*, prior to the incubation) to 9.52% in CR_C, and at the same time a decrease to 1.40% in CR_Δ. These differences between treatments can be related with the higher sensitivity of *Nitrospira* genus found in Crestuma location to salinity fluctuation. The sensitiveness of *Nitrospira* to salinity has been shown in previous studies. For instance, Bassin et al. (2011) reported that *Nitrospira* spp. disappeared in systems with elevated salinity. In accordance, Moussa et al. (2006) identified *Nitrospira* sp. as the dominant NOB within the salinity range until 10 g NaCl L^–1^. Therefore, our salinity fluctuation regime had most likely an important impact on this taxon. This is in agreement with Liu Z. et al. (2020), who performed a study on comammox communities in various mangrove ecosystems and found out that temperature and salinity were important factors shaping the comammox community. Although studies focusing on comammox species and their distribution have been emerging (Jiang et al., 2019; Zhao et al., 2019; Liu S. et al., 2020; Liu Z. et al., 2020), we still require future studies aiming toward this group of organisms to account for their actual role in the nitrification process in estuarine ecosystems.

## Conclusion

By controlling the effect of environmental factors, our microcosm experiment proved to be a good approach to assess the plasticity of estuarine AOM to different salinity regimes, showing that the presence of marked salinity gradients in estuarine systems shape communities with different functional resilience toward salinity fluctuations. The results of this study demonstrated that AOM inhabiting natural high salinity amplitude sites (our study: Afurada) displayed an elevated plasticity in terms of nitrification activity and community structure toward the incubation salinity conditions tested (constant and salinity fluctuation regimes). The AOM that inhabit the upstream sediments of the same estuarine system, that naturally experience low salinity amplitudes, were deeply affected when exposed under daily salinity fluctuation conditions. Our results suggested that *Nitrosomonas* and “*Ca.* Nitrosopumilus” have a higher influence on the nitrification process in downstream sediments, while *Nitrospira*, “*Ca.* Nitrosoarchaeum” and “*Ca.* Nitrosotenuis” genus displayed an important role on the nitrification process in the upstream sediments. Specific studies that target *Nitrospira* phylotypes including comammox are required to understand their functional contribution in estuarine systems. Finally, further research focusing on the adaptation of AOM toward salinity regimes are required to improve our understanding on the mechanisms that control the selection of this group of prokaryotes and their contributions to estuarine nitrification budgets.

## Data Availability Statement

The datasets presented in this study can be found in online repositories. The names of the repository/repositories and accession number(s) can be found below: https://www.ebi.ac.uk/ena, PRJEB38114.

## Author Contributions

JS and CM designed the study. JS performed the experiments, analyzed the samples, and wrote the manuscript. JS and AS analyzed the data and produced plots. AS, HR, and CM assisted in writing the manuscript. AS did the bioinformatic analysis. CM provided conceptual contributions to the experiments. All authors contributed conceptually and reviewed the manuscript.

## Conflict of Interest

The authors declare that the research was conducted in the absence of any commercial or financial relationships that could be construed as a potential conflict of interest.
