# Supplementary material for: The Response of Estuarine Ammonia-Oxidizing Communities to Constant and Fluctuating Salinity Regimes
Source: Front Microbiol. 2020 Nov 26;11:574815. doi: 10.3389/fmicb.2020.574815 (PMC7727400; doi:10.3389/fmicb.2020.574815)
Supplement: Supplementary file 1 [file Data_Sheet_1.docx]

**The response of estuarine ammonia-oxidizing communities to constant and fluctuating salinity regimes**

J.P. Santos^1,2 *^, A.G.G. Sousa^1^, H. Ribeiro^1,3^ and C. Magalhães^1,4,5,6^

^1^ CIIMAR - Centro Interdisciplinar de Investigação Marinha e Ambiental, Universidade do Porto, Terminal de Cruzeiros do Porto de Leixões, Av. General Norton de Matos s/n, 4450-208, Matosinhos, Portugal

^2^ Department F.A. Forel for Environmental and Aquatic Sciences, Section of Earth and Environmental Sciences and Institute for Environmental Sciences, University of Geneva, Carl-Vogt 66, CH-1211 Geneva, Switzerland

^3^ Abel Salazar Institute of Biomedical Sciences, University of Porto (ICBAS-UP), Rua Jorge Viterbo Ferreira, 228, Porto 4050-313, Portugal

^4^ FCUP - Faculdade de Ciências da Universidade do Porto, Porto, Portugal

^5^ School of Science & Engineering, University of Waikato, Hamilton, New Zealand

^6^ Ocean Frontier Institute, Dalhousie University, Halitax, NS, Canada

**Correspond author:**

*Corresponding author: joaofs21@gmail.com

Supplementary Figures


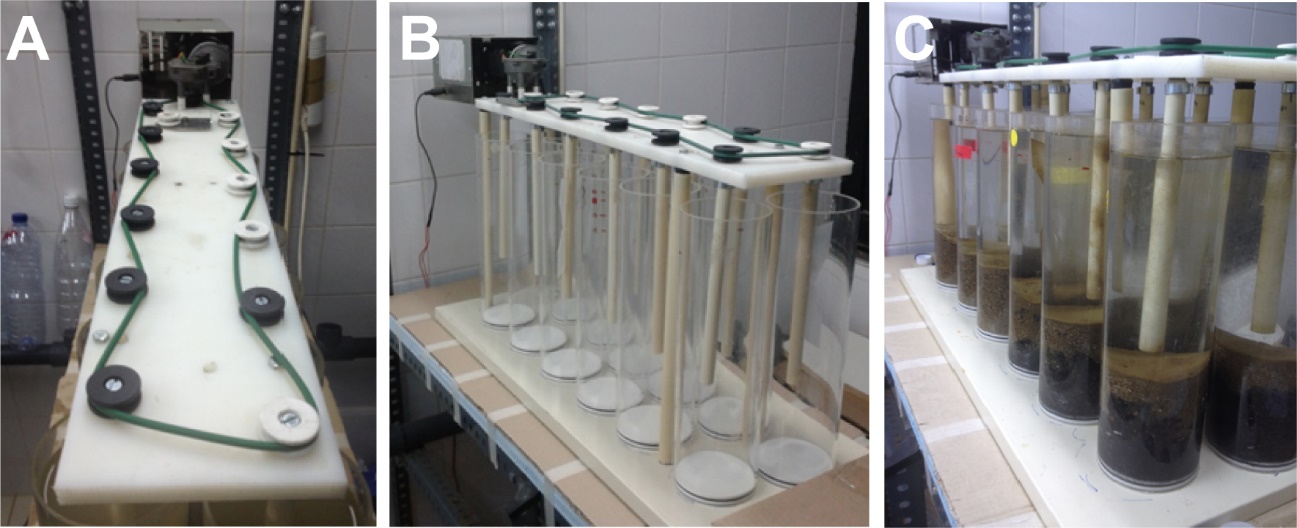


**Figure S1.** Pictures of the upper part (**Figure S1-A**) of the autonomous water agitation system for 12 acrylic reactors before (**Figure S1-B**) and during the incubation (**Figure S1-C**) experiment.


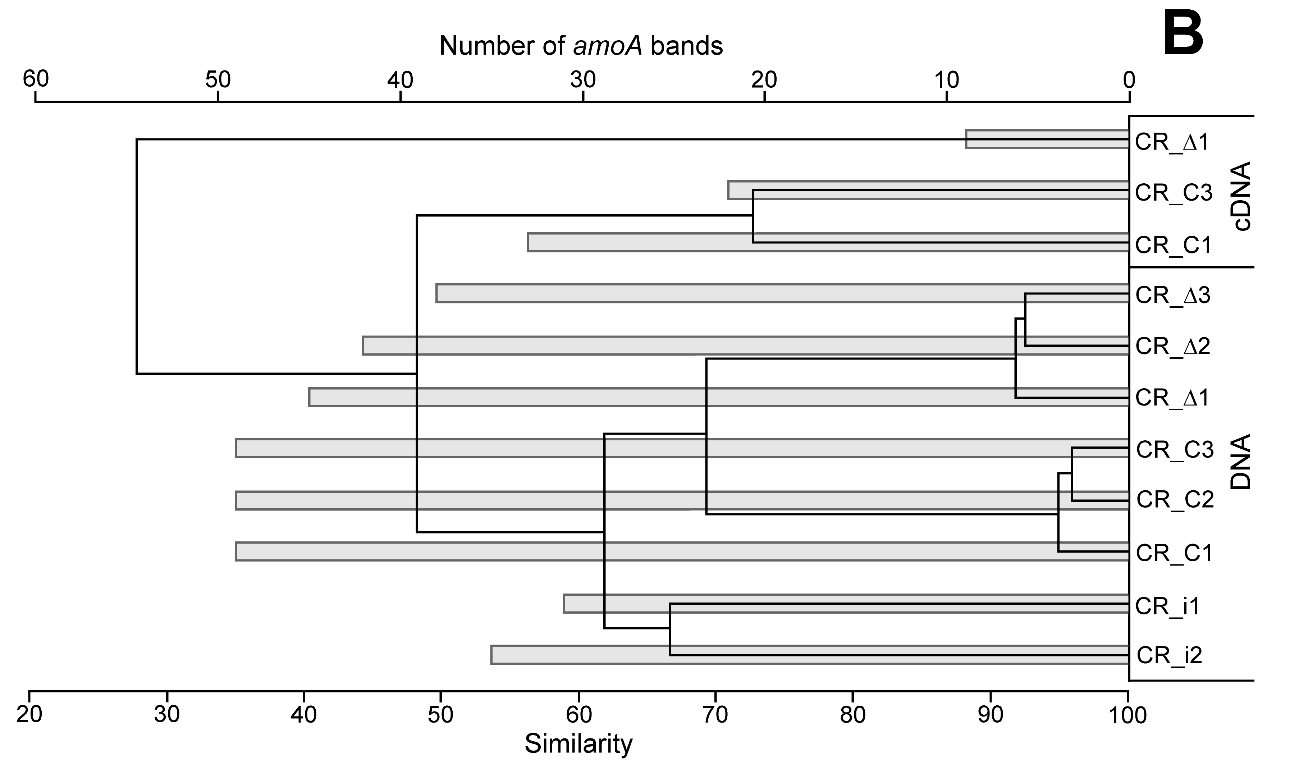

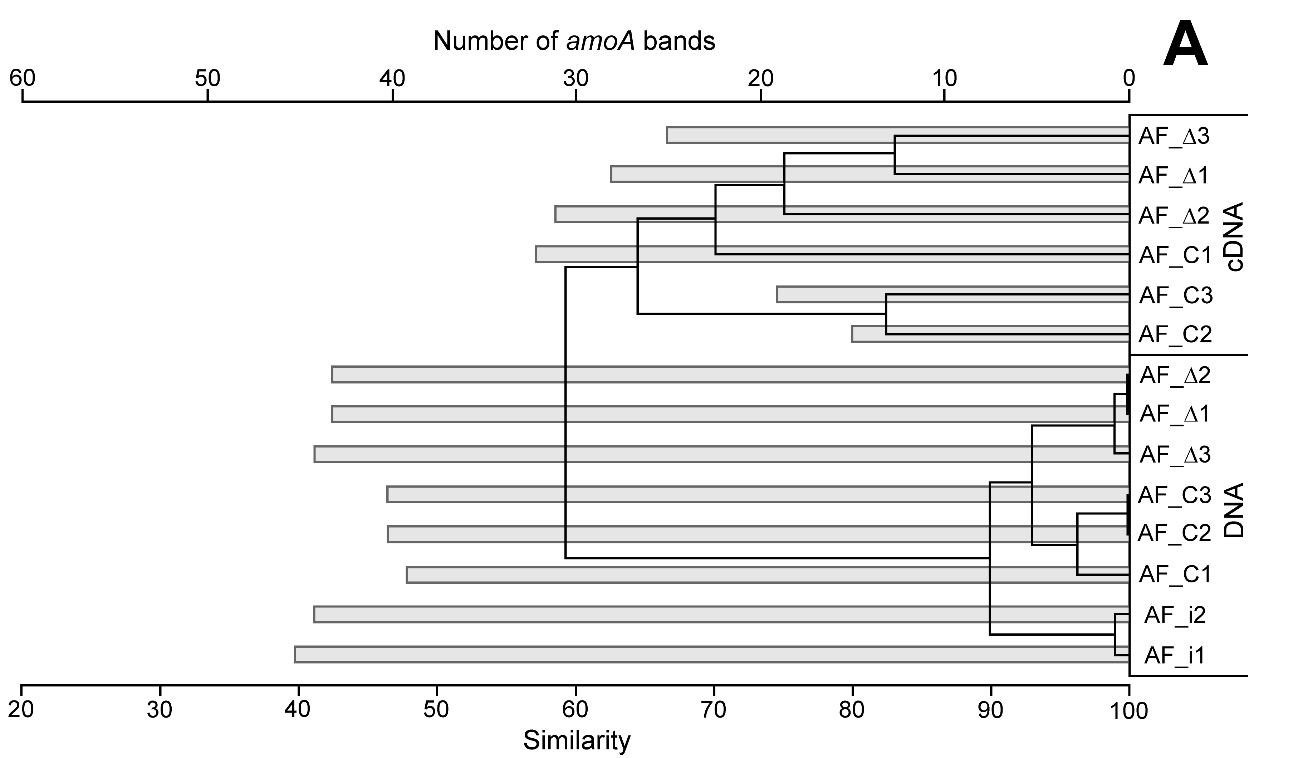
**Figure S2.** Hierarchical cluster analysis using the average linkage of Bray-Curtis similarity for the presence or absence of archaeal *amoA* transcripts (cDNA) and genes (DNA) under different salinity treatments (constant – CR_C and AF_C, and fluctuation – CR_Δ and AF_Δ) from Afurada (**Figure S2-A**) and Crestuma (**Figure S2-B**) sediments. Archaeal *amoA* genes include initial *in situ* sediment (AF_i and CR_i samples). The relative “richness” of *amoA* transcripts and genes is given based on the number of bands of each PCR-DGGE and RT-PCR-DGGE profile generated (gray bars).


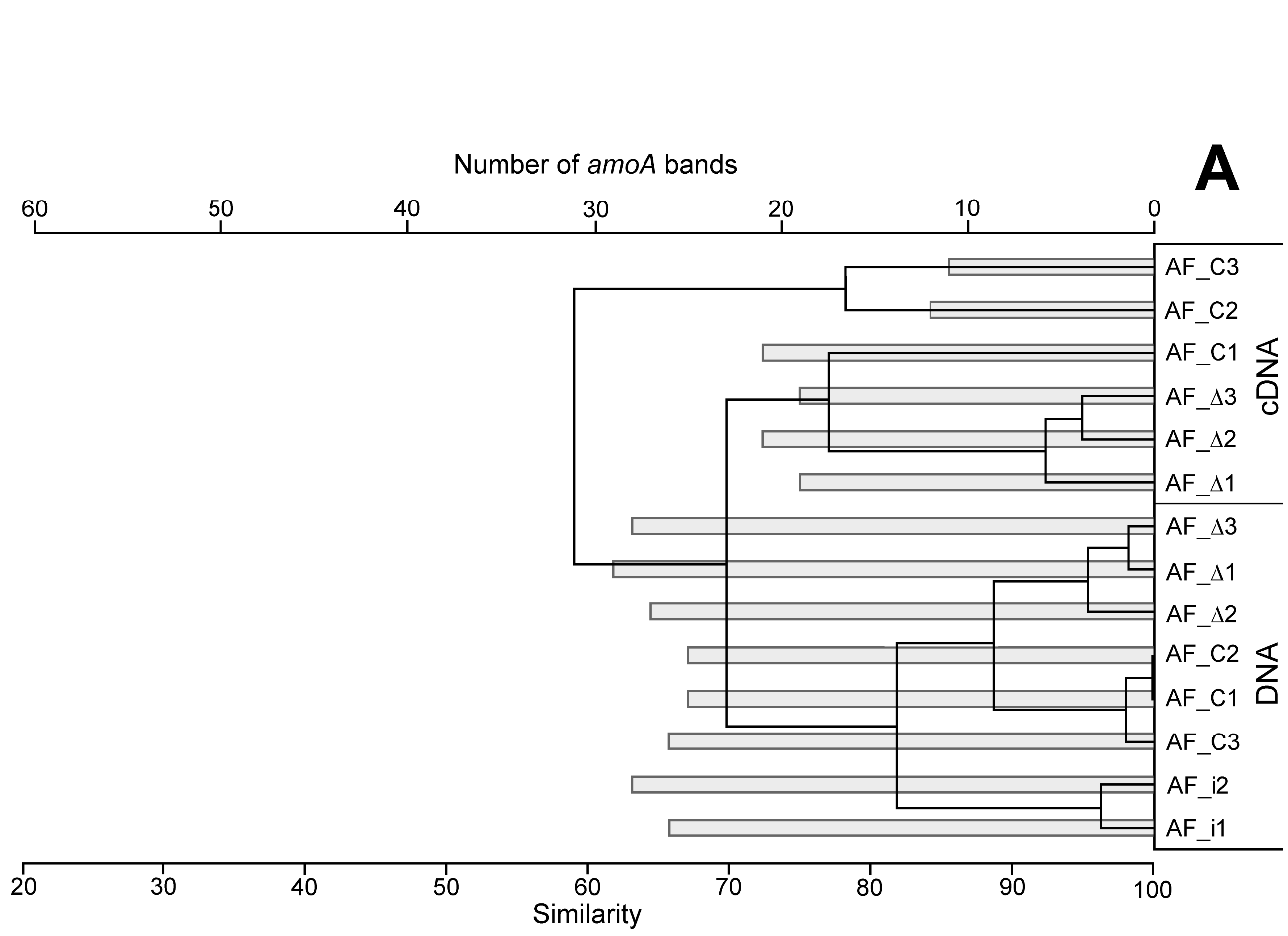

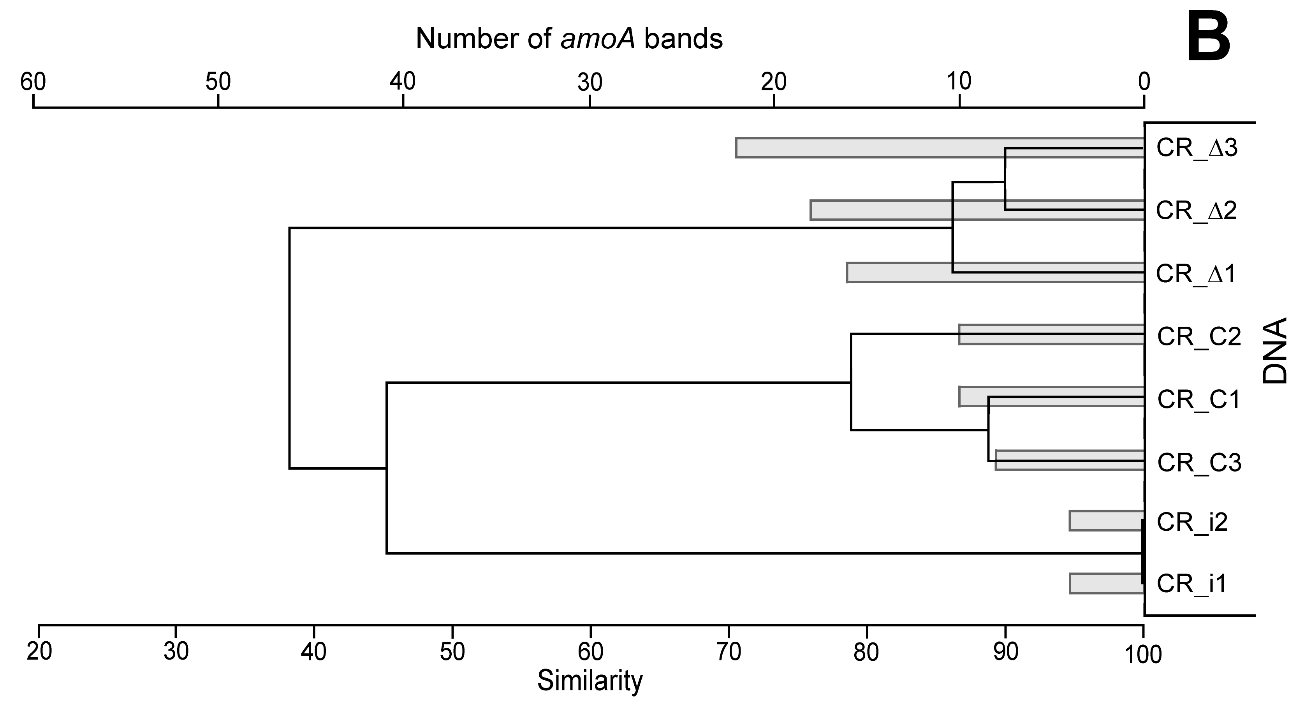


**Figure S3.** Hierarchical cluster analysis using the average linkage of Bray Curtis similarities for the presence or absence of *β-*proteobacterial *amoA* transcripts (cDNA) and genes (DNA) under different salinity treatments (constant – CR_C and AF_C, and fluctuation – CR_Δ and AF_Δ) from Afurada (**Figure S3-A**) sediments and only proteobacterial *amoA* genes for Crestuma (**Figure S3-B**) sediments. Proteobacterial *amoA* genes include initial *in situ* sediment (AF_i and CR_i samples). The relative “richness” of *amoA* transcripts and genes is given based on the number of bands of each PCR-DGGE and RT-PCR DGGE profile generated (gray bars).


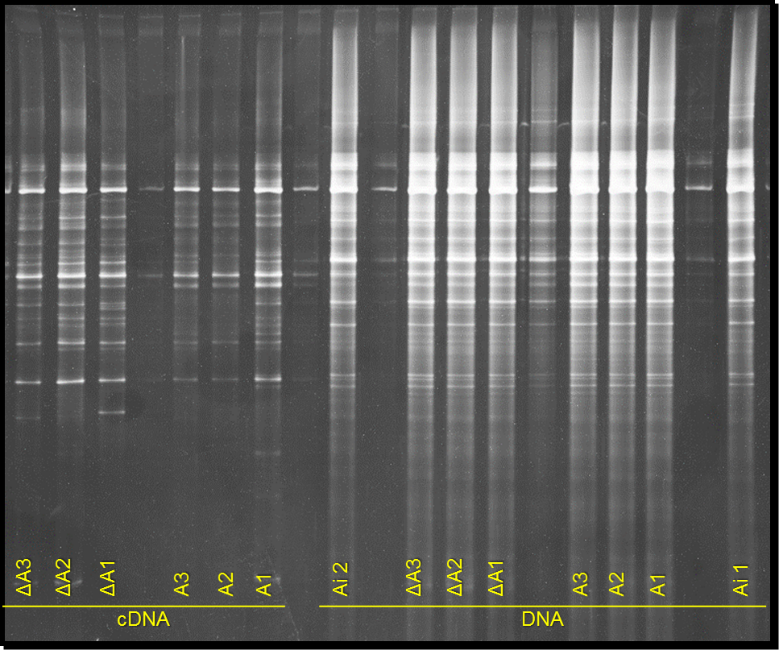


**AF_Δ1**

**AF_Δ2**

**AF_Δ3**

**AF_C3**

**AF_C2**

**AF_C1**

**AF_i2**

**AF_C3**

**AF_C2**

**AF_C1**

**AF_Δ1**

**AF_Δ2**

**AF_Δ3**

**AF_i1**

**Figure S4.** Image of the DGGE gel of *amoA* gene fragments and transcripts amplified from ammonia-oxidizing archaea on Afurada sediment samples exposed to different salinity conditions (constant salinity (AF_C1, AF_C2 and AF_C3) and fluctuation salinity (AF_ Δ1, AF_Δ2 and AF_Δ3)) as well initial Afurada sediment (AF_i1 and AF_i2).


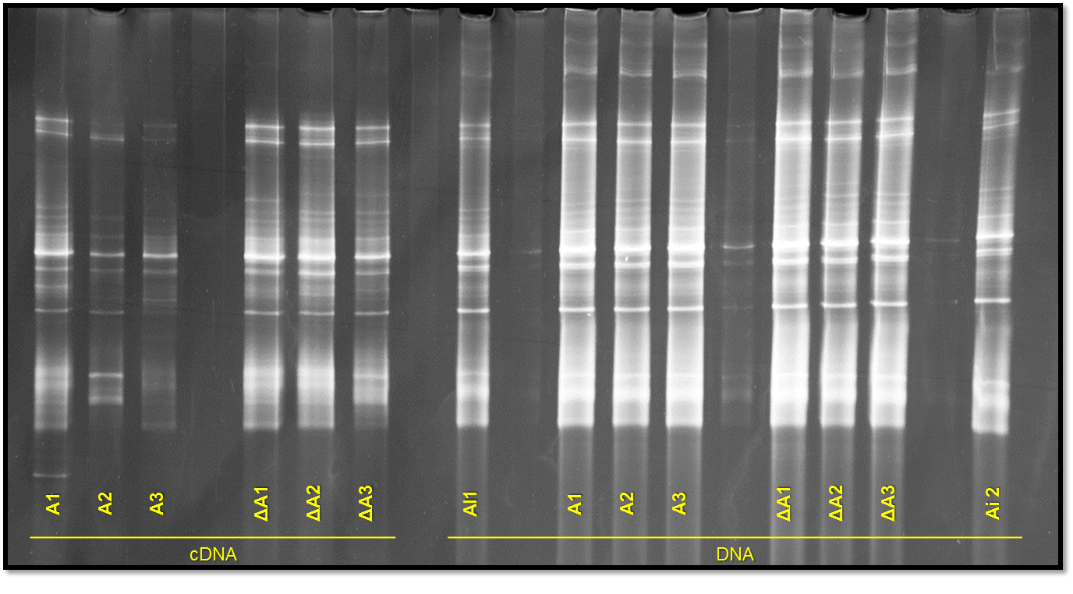


**AF_C1**

**AF_C2**

**AF_C3**

**AF_ Δ 1**

**AF_ Δ 2**

**AF_ Δ 3**

**AF_i1**

**AF_ Δ 1**

**AF_ Δ 2**

**AF_ Δ3**

**AF_C3**

**AF_C2**

**AF_C1**

**AF_i2**

**Figure S5.** Image of the DGGE gel of *amoA* gene fragments and transcripts amplified from ammonia-oxidizing bacteria on Afurada sediment samples exposed to different salinity conditions (constant salinity (AF_C1, AF_C2 and AF_C3) and fluctuation salinity (AF_Δ1, AF_Δ2 and AF_Δ3)) as well initial Afurada sediment (AF_i1 and AF_i2).


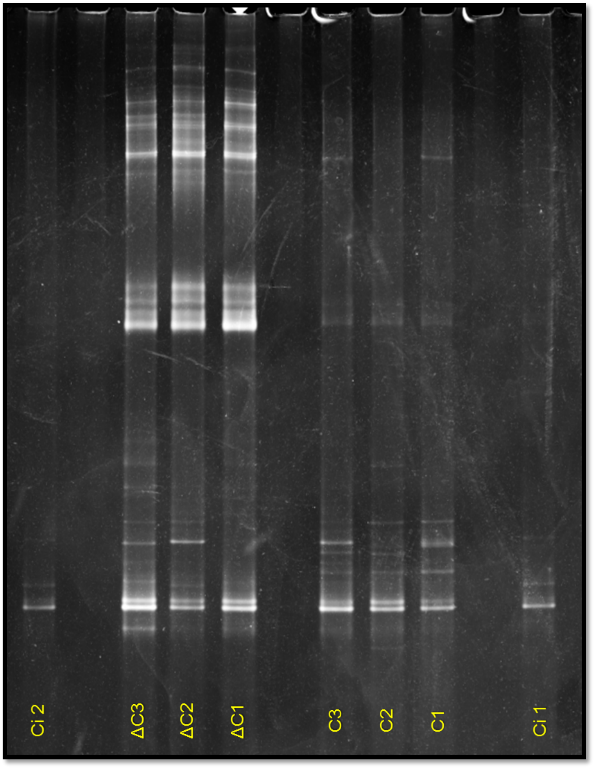


**B**

**CR_i1**

**CR_i2**

**CR_C1**

**CR_C2**

**CR_C3**

**CR_ Δ3**

**CR_ Δ2**

**CR_ Δ1**

**DNA**


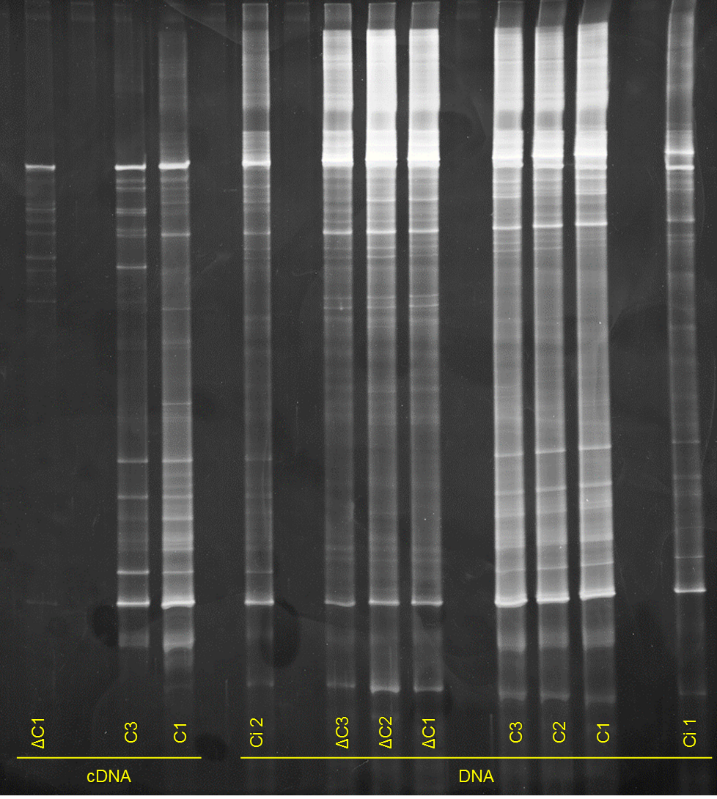


**CR_C1**

**CR_C3**

**CR_i2**

**CR_C1**

**CR_C2**

**CR_C3**

**CR_i1**

**CR_ Δ 2**

**CR_ Δ3**

**CR_ Δ1**

**CR_ Δ1**

**A**

**Figure S6.** Image of the DGGE gel of *amoA* gene fragments and transcripts (**Figure S6-A**) amplified from ammonia-oxidizing archaea on Crestuma sediment samples exposed to different salinity conditions (constant salinity (CR_1, CR_2 and CR_3) and fluctuation salinity (CR_Δ1, CR_Δ2 and CR_Δ3)) as well initial Crestuma sediment (CR_i1 and CR_i2). Image of the DGGE gel of *amoA* gene fragments (**Figure S6-B**) amplified from ammonia-oxidizing bacteria on Crestuma sediment samples exposed to different salinity conditions (constant salinity (CR_1, CR_2 and CR_3) and fluctuation salinity (CR_Δ1, CR_Δ2 and CR_Δ3)) as well initial Crestuma sediment (CR_i1 and CR_i2).


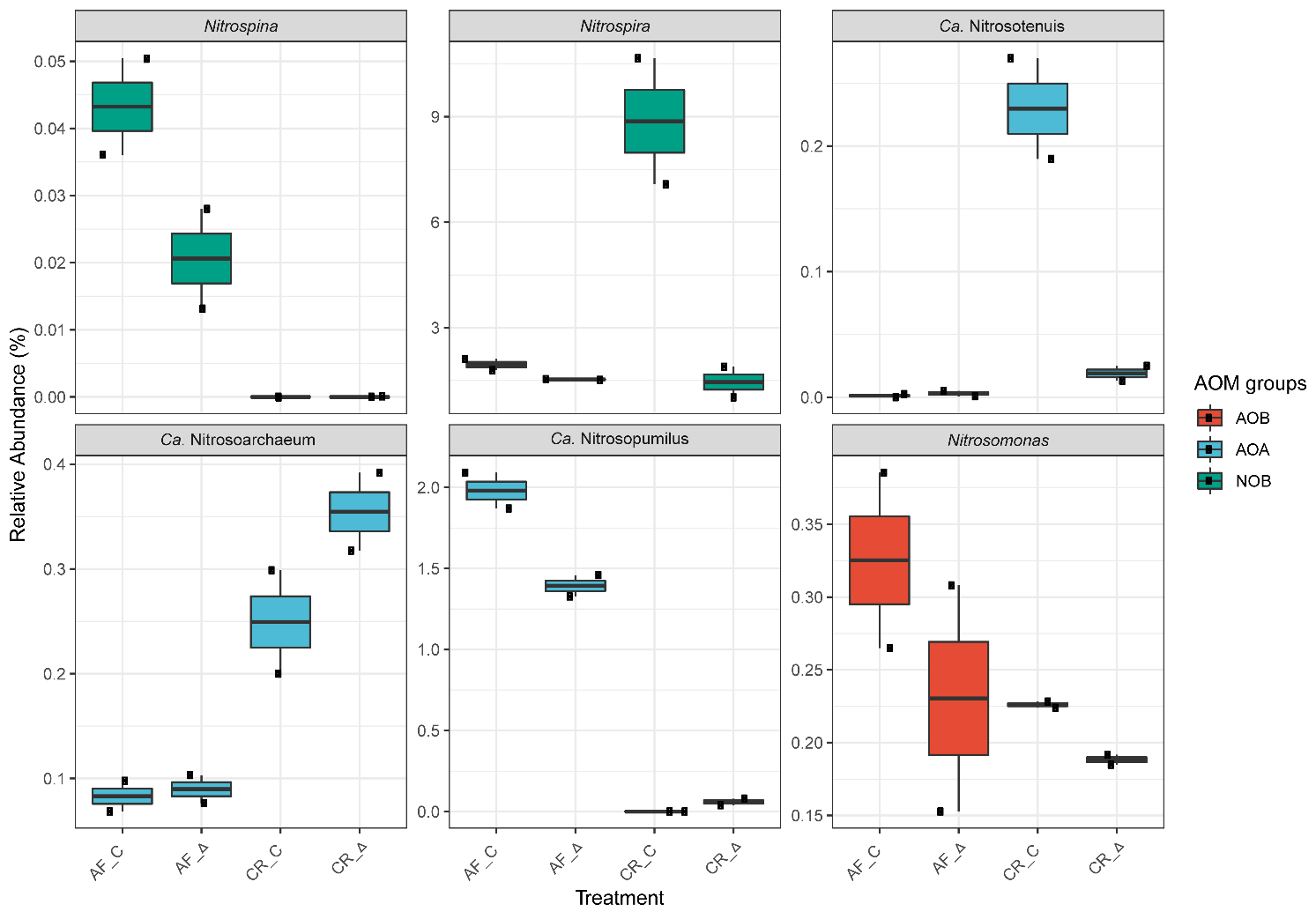


**Figure S7.** Box plot representing the average relative abundance of the different ammonia-oxidizing genera identified in the different salinity treatments (constant – AF_C and CR_C, and fluctuation – AF_Δ and CR_Δ; n=2).

Supplementary Tables

**Table S1.** Salinity and temperature mean values from 36 days of daily monitoring in constant salinity treatments reactors from Afurada (AF_C_1_, AF_C_2_ and AF_C_3_) and Crestuma (CR_C_1_, CR_C_2_ and CR_C_3_) and in fluctuation salinity reactors from Afurada (AF_Δ_1_, AF_Δ_2_ and AF_Δ_3_) and Crestuma (CR_Δ_1_, CR_Δ_2_ and CR_Δ_3_).

| **Reactor** | **Number of water  renewal’s** | **Salinity (psu)** | | | | **Temperature (°C)** | |
| --- | --- | --- | --- | --- | --- | --- | --- |
|  |  | **Mean** | **Min** | **Max** | **SD** | **Mean** | **SD** |
| AF_C_1_ | 69 | 15.3 | 15.0 | 16.1 | 0.2 | 18.8 | 2.2 |
| AF_C_2_ | 69 | 15.3 | 15.0 | 16.1 | 0.2 | 18.8 | 2.2 |
| AF_C_3_ | 69 | 15.3 | 15.0 | 16.1 | 0.2 | 18.8 | 2.2 |
| AF_∆_1_ | 69 | 15.5 | 1.5 | 29.2 | 9.4 | 18.8 | 2.2 |
| AF_∆_2_ | 69 | 15.5 | 1.5 | 29.3 | 9.5 | 18.8 | 2.2 |
| AF_∆_3_ | 69 | 15.5 | 1.5 | 29.1 | 9.4 | 18.8 | 2.2 |
| CR_C_1_ | 69 | 0.2 | 0.1 | 1.7 | 0.2 | 18.8 | 2.2 |
| CR_C_2_ | 69 | 0.2 | 0.1 | 1.4 | 0.2 | 18.8 | 2.2 |
| CR_C_3_ | 69 | 0.1 | 0.1 | 1.3 | 0.2 | 18.8 | 2.2 |
| CR_∆_1_ | 69 | 15.5 | 1.4 | 29.3 | 9.5 | 18.8 | 2.2 |
| CR_∆_2_ | 69 | 15.4 | 1.3 | 29.3 | 9.5 | 18.8 | 2.2 |
| CR_∆_3_ | 69 | 15.5 | 1.2 | 29.3 | 9.6 | 18.8 | 2.2 |

| **Site** | **Incubation condition** | **ID** | **µmol N mL^-1^ cm^-2^ h^-1^** |
| --- | --- | --- | --- |
| Afurada | Constant salinity | AF_C1 | 0.0048 |
|  |  | AF_C2 | 0.0036 |
|  |  | AF_C3 | 0.0036 |
|  | Salinity fluctuation | AF_Δ1 | 0.0033 |
|  |  | AF_Δ2 | 0.0035 |
|  |  | AF_Δ3 | 0.0040 |
| Crestuma | Constant salinity | CR_C1 | 0.0048 |
|  |  | CR_C2 | 0.0062 |
|  |  | CR_C3 | 0.0055 |
|  | Salinity fluctuation | CR_Δ1 | 0.0014 |
|  |  | CR_Δ2 | N/A |
|  |  | CR_Δ3 | 0.0013 |

**Table S2**. Potential nitrification rates ([^15^N]O_2_^-^ + [^15^N]O_3_^-^ produced) in triplicate treatments after the addition of 20 µM of [^15^N]H_4_^+^ in Afurada (AF_C and and AF_Δ) and Crestuma (CF_C and and CR_Δ) reactors. N/A – not available.

**Table S3**. Potential nitrification rates ([^15^N]O_2_^-^ + [^15^N]O_3_^-^ produced) measured in triplicate slurries after the addition of 100 µM of [^15^N]H_4_^+^ in Afurada (AF_C and and AF_Δ) and Crestuma (CF_C and and CR_Δ) salinity treatments.

| **Site** | **Incubation condition** | **ID** | **µmol N mL^-1^ g^-1^ h^-1^** |
| --- | --- | --- | --- |
| Afurada | Constant salinity | AF_C1 | 0.0027 |
|  |  | AF_C2 | 0.0029 |
|  |  | AF_C3 | 0.0030 |
|  | Salinity fluctuation | AF_Δ1 | 0.0051 |
|  |  | AF_Δ2 | 0.0029 |
|  |  | AF_Δ3 | 0.0024 |
| Crestuma | Constant salinity | CR_C1 | 0.0035 |
|  |  | CR_C2 | 0.0027 |
|  |  | CR_C3 | 0.0028 |
|  | Salinity fluctuation | CR_Δ1 | 0.0014 |
|  |  | CR_Δ2 | 0.0012 |
|  |  | CR_Δ3 | 0.0013 |

**Table S4.** Relative abundance (%) of the top 20 dominant phyla in downstream (AF) and upstream (CR) *in situ* sediments (AF_i, CR_i) and laboratory experiments (AF_C, AF_Δ, CR_C and CR_Δ). The values correspond to the relative abundance of the two replicates (1 and 2) analyzed per each salinity treatment (CR_C, CR_Δ, AF_C and AF_Δ).

| **Phylum** | **Relative abundance (%) in the different treatments** | | | | | | | | | |
| --- | --- | --- | --- | --- | --- | --- | --- | --- | --- | --- |
|  | **AF_i** | **AF_C1** | **AF_C2** | **CR_i** | **CR_C2** | **CR_C3** | **AF_Δ1** | **AF_ Δ2** | **CR_ Δ1** | **CR_Δ2** |
| *Proteobacteria* | 39.5 | 47.2 | 50.1 | 41.4 | 45.9 | 46.3 | 45.9 | 53.4 | 70.8 | 61.9 |
| *Bacteroidetes* | 31.0 | 12.5 | 14.5 | 23.5 | 15.3 | 13.8 | 12.8 | 13.5 | 14.3 | 15.7 |
| *Planctomycetes* | 3.7 | 12.5 | 8.5 | 7.6 | 9.6 | 9.2 | 13.6 | 7.2 | 6.1 | 7.6 |
| *Cyanobacteria* | 14.8 | 7.2 | 6.7 | 5.7 | 1.0 | 0.7 | 14.9 | 9.1 | 0.8 | 0.6 |
| *Acidobacteria* | 1.7 | 5.2 | 4.7 | 5.7 | 7.9 | 7.6 | 2.0 | 3.8 | 1.5 | 3.1 |
| *Nitrospirae* | 1.0 | 1.8 | 2.1 | 3.0 | 7.1 | 10.7 | 1.5 | 1.5 | 1.0 | 1.9 |
| *Verrucomicrobia* | 2.0 | 0.7 | 0.8 | 5.0 | 3.8 | 3.2 | 0.5 | 0.8 | 0.7 | 1.6 |
| *Thaumarchaeota* | 2.3 | 3.7 | 3.2 | 0.4 | 0.4 | 0.7 | 3.0 | 2.4 | 0.5 | 0.4 |
| *Gemmatimonadetes* | 0.3 | 1.3 | 1.1 | 2.7 | 3.0 | 2.1 | 0.6 | 1.0 | 2.2 | 3.3 |
| *Chloroflexi* | 1.4 | 2.1 | 2.1 | 1.1 | 1.7 | 1.9 | 1.9 | 2.4 | 0.3 | 0.9 |
| *Actinobacteria* | 0.9 | 1.4 | 1.3 | 1.0 | 1.0 | 0.8 | 1.0 | 1.5 | 0.4 | 1.2 |
| *Latescibacteria* | 0.3 | 1.1 | 1.0 | 0.3 | 1.0 | 1.0 | 0.4 | 0.8 | 0.2 | 0.3 |
| *Armatimonadetes* | 0.1 | 0.0 | 0.0 | 0.9 | 0.5 | 0.5 | 0.0 | 0.0 | 0.1 | 0.2 |
| *Firmicutes* | 0.2 | 0.4 | 0.5 | 0.1 | 0.1 | 0.0 | 0.2 | 0.3 | 0.0 | 0.0 |
| *Spirochaetes* | 0.2 | 0.2 | 0.2 | 0.1 | 0.1 | 0.1 | 0.1 | 0.3 | 0.0 | 0.0 |
| *Lentisphaerae* | 0.1 | 0.1 | 0.2 | 0.0 | 0.0 | 0.0 | 0.1 | 0.1 | 0.3 | 0.3 |
| *Kiritimatiellaeota* | 0.1 | 0.3 | 0.4 | 0.1 | 0.0 | 0.0 | 0.2 | 0.3 | 0.0 | 0.0 |
| *Deinococcus-Thermus* | 0.1 | 0.1 | 0.1 | 0.3 | 0.1 | 0.1 | 0.0 | 0.0 | 0.1 | 0.1 |
| *Rokubacteria* | 0.0 | 0.0 | 0.0 | 0.2 | 0.1 | 0.2 | 0.0 | 0.0 | 0.1 | 0.2 |
| *Omnitrophicaeota* | 0.0 | 0.1 | 0.1 | 0.1 | 0.3 | 0.2 | 0.0 | 0.1 | 0.0 | 0.0 |
| Other | 0.5 | 2.1 | 2.4 | 0.7 | 1.3 | 1.1 | 1.2 | 1.5 | 0.3 | 0.6 |

**Table S5.** Relative abundance (%) of the nitrifying genera found in downstream (AF) and upstream (CR) *in situ* sediments (AF_i, CR_i) and laboratory experiments (AF_C, AF_Δ, CR_C and CR_Δ). The values correspond to the relative abundance of the two replicates (1 and 2) analyzed per each salinity treatment (CR_C, CR_Δ, AF_C and AF_Δ).

| **Group** | **Family** | **Genus** | **Relative abundance (%) in the different treatments** | | | | | | | | | |
| --- | --- | --- | --- | --- | --- | --- | --- | --- | --- | --- | --- | --- |
|  |  |  | **AF_i** | **AF_C1** | **AF_C2** | **CR_i** | **CR_C2** | **CR_C3** | **AF_Δ1** | **AF_Δ2** | **CR_C1** | **CR_C2** |
| **AOB** | Nitrosomonadaceae | *Nitrosomonas* | 0.2 | 0.3 | 0.4 | 0.0 | 0.2 | 0.2 | 0.2 | 0.3 | 0.2 | 0.2 |
| **AOA** | Nitrosopumilaceae | "*Ca*. Nitrosotenuis" | 0.0 | 0.0 | 0.0 | 0.1 | 0.2 | 0.3 | 8.8 | 0.0 | 0.0 | 0.0 |
|  |  | "*Ca*. Nitrosopumilus" | 0.8 | 2.1 | 1.9 | 0.0 | 0.0 | 0.0 | 1.5 | 1.3 | 0.1 | 0.0 |
|  |  | "*Ca*. Nitrosoarchaeum" | 0.1 | 0.1 | 0.1 | 0.2 | 0.2 | 0.3 | 0.1 | 0.1 | 0.4 | 0.3 |
| **NOB** | Nitrospiraceae | *Nitrospina* | 0.0 | 0.0 | 0.1 | 0.0 | 0.0 | 0.0 | 0.0 | 0.0 | 0.0 | 0.0 |
|  |  | *Nitrospira* | 1.0 | 1.8 | 2.1 | 3.0 | 7.1 | 10.7 | 1.5 | 1.5 | 1.0 | 1.9 |
